# Worked to Death

**Published:** 1871-07

**Authors:** 


### WORKED TO DEATH.

Horace Greeley is perhaps the hardest worked man, mentally, in the United States; this has been continued for a score of years; yet he is fat and healthy to-day, of genial temper, and to all appearance, likely to remain so for several decades to come.

A brother editor worked quite as hard; his mental labors were at times herculean; but he died in a moment, while he should have been in the very highest condition of mental capabilities, — died ten years younger than Mr. Greeley.

Within a week it is written of a man who died "much under thirty," that he was one of the ablest young men of the younger generation. He was the general editor of a daily paper, its dramatic critic, and one of its leading editorial writers, all at the same time. He was the correspondent of a New York and Chicago paper, his letters being "models of critical correspondence;" besides, he wrote for a weekly pictorial, and for three of the leading monthlies of the nation. When he died, he had accomplished "much more than most men of twice his age."

Mental labor is as much a promoter of long life as physical labor; hard thinking does as much to insure bodily health as hard work.

HARD WORK KILLS

Prematurely, always; hard working men, such as spend most of their working time out-of-doors, die, on an average, from ten to twenty years sooner than hard students. It is not the hard thinking, nor the hard working which causes the premature death of some who work hard and think hard. It should be known more generally among all classes, that

HARD EATING KILLS

Just as certainly and as soon, yes, sooner, than hard drinking. Horace Greeley has always lived temperately and regularly; so has William Cullen Bryant, now fourscore; the brother editor who died was a gourmand, a drunkard, and a debauchee. Count Cavour, without a superior in his line, died of a dinner; he was an habitual glutton; the cares of state never killed him. We do not hear that Newton had dyspepsia, that Galileo died of exhaustion.

Hard work will not give long life to a man unless he lives regularly, temperately, and has abundant sleep; the hardest student in the world will live long, if he eats regularly of plain food, sleeps well, and keeps the bodily functions in regular working order. Living up to the natural laws, both hard working and hard thinking will carry men to a cheerful and hale fourscore.

